# Mapping Interactions among Green Innovations Barriers in Manufacturing Industry Using Hybrid Methodology: Insights from a Developing Country

**DOI:** 10.3390/ijerph18157885

**Published:** 2021-07-26

**Authors:** Sajid Ullah, Naveed Ahmad, Farman Ullah Khan, Alina Badulescu, Daniel Badulescu

**Affiliations:** 1 School of Economics and Management, Xi’an University of Technology, Xi’an 710048, China; sajidkhan6695@stu.xaut.edu.cn; 2 School of Management, Northwestern Polytechnical University, Xi’an 710072, China; naveedahmad@mail.nwpu.edu.cn; 3 Department of Business Administration, Lahore Leeds University, Lahore 54000, Pakistan; 4 School of Management, Xi’an Jiaotong University, Xi’an 710000, China; 5 Department of Economics and Business, Faculty of Economic Sciences, University of Oradea, 410087 Oradea, Romania; dbadulescu@uoradea.ro

**Keywords:** green innovations, barriers, developing countries, ISM-MICMAC modeling, manufacturing industry, Pakistan

## Abstract

Recent years have witnessed continuous rise in adopting green innovations which is considered as an important organizational instrument to achieve profits by reducing environmental deterioration. However, green innovation in developing countries, especially in Pakistan, is surprisingly scant as compared to developed countries. This paper empirically investigated obstacles to green innovations in Pakistani manufacturing firms. Specifically, a novel three phase methodological framework was applied to investigate significant barriers and filtration by integrating Delphi method (DM), interpretive structural modeling (ISM), and cross-impact matrix multiplication applied to classification (MICMAC). Our results highlighted that lack of enforceable laws regarding returned goods and recycled products, lack of rules and regulations for green practices, and lack of collaboration with government and environmental institutions are most critical barriers. However, fear of failure about green innovation is least important barriers to green innovations adoption. This study offers interesting clues to promote green innovation in manufacturing industry.

## 1. Introduction

Nowadays, customers are paying much attention to the environment than ever before [1]. Governments are also formulating tight rules and regulations to control the environmental pollution created by the industrial sector [2]. Firms are under pressure to address sustainability issues, specifically poor environmental reputations [3]. Manufacturing industry is the backbone to country’s development, and their environmental harms cannot be unnoticed because of the larger production system and continuous operations. These larger manufacturing firms should inevitably indulge in green innovations due to continuous production and process [4]. Considering this, manufacturing firms seek environmental practices to protect the environment and simultaneously build up a good reputation [5]. One approach towards ‘going green’ is the implementation of green innovations through which firms can contribute to sustainable development and mitigate environmental hazards and costs [6]. Kemp [7] defined green innovation as the “process, technique, product development or service, management and business method that is novel to the firm during production throughout its lifecycle, which results in mitigation of environmental pollution and risks and other harmful impacts on resources, including the use of energy”.

Advanced and more developed countries have abundant resources for taking precautionary measures and enforcing laws to control environmental harms [8]. The US and European Union (EU) have a specific set of broad environmental laws that play a central role in doing business [9]. However, Africa and Asia have significant environmental concerns, and these countries are emerging economies, where large production and processing is taking place [10]. While, green innovation is adopted through environmental regulations and resources, which can improve firm performance [11]. There is an intense need to address environmental issues, especially in emerging economies that may be possible through corporate green innovation. Companies all over the world embrace green innovation practices when such initiatives are found to be competitive, explore new market opportunities, increase profit, achieve operational success, and improve environmental performance [12,13,14]. Also, green innovation is considered a viable environmental policy for manufacturing firms to attain both ecological and financial benefits [15,16]. However, adopting a green innovation is often marred with certain barriers. Manufacturing firms are at the back foot when it comes to implementation of green practices. Manufacturing firms fail in implementing green innovation process as they usually face obstacles regarding bringing new ideas regarding product or process innovation. No doubt, green innovation implementation success depends on a firm’s ability to control green innovation barriers [17]. Addressing barriers in green innovation, specifically among manufacturing firms, is important for twofold reasons. First, innovation policy is required to address systematic failures and know why firms are excluded from the innovation contest [18,19]. Second, investigating general hindrances faced by firms towards green innovation is utterly important, especially in case those fail in launching a new market or product [20].

Despite the great importance being given to sustainable practices in manufacturing firms, the practices of green concepts have not widely implemented in developing countries [21]. Green initiatives are still in their infancy in developing countries, especially in Pakistan due to certain barriers. Out of 98% manufacturing units, 80% are located in the urban areas; however, most of the units have no sufficient control over greenhouse gas (GHG) emission [22]. Actually, many past studies on green innovation/green practices have focused on developed countries (Europe and North America), and Australia [23,24,25,26] but far less attention has paid to Southeast Asian developing economies. In addition, the lack of theoretical and empirical literature in the manufacturing industry is scarce. Logically, the generalization of impediments to the adoption of green innovation cannot equally be applied to the second-largest economy in South Asia, Pakistan, due to certain reasons: massive cultural clash, firm size, the difference in economic growth, different rules and regulations, and corporate mindset. Addressing barriers to green innovation adoption is mandatory that could help in initiating green innovation practices in the manufacturing industry.

Therefore, this research grasps ideas with proper guidance from academicians, state regulators, and professional bodies to identify significant barriers and establish policies to mitigate these barriers. Such practices will ensure that manufacturing industries in a developing country, like Pakistan, thoroughly follow the green innovation in the manufacturing process. Moreover, this study will help industries in exploring barriers that require awareness and those that need more attention for successful green innovation implementation.

Considering the wake-up call for manufacturing firms to address green innovation barriers, this research covers these objectives:

- Exploring green innovation barriers through comprehensive literature review;
- Developing a contextual relationship between different barriers to green innovation;
- Determining the driving and dependence power of barriers to green innovation.

The remaining paper is structured as follows. Section 2 presents a detailed literature review on green innovation, green innovation barriers, and a Pakistan manufacturing perspective. Section 3 describes the research methodology and framework by highlighting the main steps that consisted of our research. Section 4 illustrates the results and discussion, while Section 5 and Section 6 presents policy, comparative study, managerial implications and conclusion respectively.

## 2. Literature Review

### 2.1. Green Innovation in Current Era

A stream of literature witness sustainability and other related practices—such as CSR, corporate citizenship, and corporate social performance—as strategic priorities for all companies in various industries and countries [27,28]. One of the crucial challenges being faced by firms is environmental protection, so many firms are engaged in adopting green innovation policies to achieve sustainable development. Therefore, a growing number of firms have pursued green innovation to gain balanced economic growth and sustainable development [29]. Environmental degradation is one of the biggest challenges in the world. As a major driver to the economic development of a country, the manufacturing sector is one of the significant contributors to such degradation [30]. These environmental issues are real challenges for developing countries that need to be remedied. For example, Eckersley [31] states that in developing countries, firms would focus more on adopting green innovation practices and advanced technology to innovate new products as necessary that are eco-friendly, such as imposing a ban on plastic bags and replace it with biodegradable plastic bags. The manufacturing industries have increased the consumption of resources as industrial growth emerges [32]. This situation pulled the supply side of resources and dragged them into natural degradation. Furthermore, it has an overall bad effect on the global environment giving rise to climate change and global warming [33]. Although environmentalists have addressed these concerns for better operations of manufacturing organizations and titled green thinking to reap the benefits in the shape of environmental and economic sustainability [34]. However, our study aims to find out barriers of green innovation challenges that caters to renewable and natural resources, waste handling, clean air and water, and emission reduction in hazardous gas [35].

Green and sustainable development are two main pillars of organizations to achieve sustainability goals of 2030. From environmental points of view, the word ‘green’ refers to those products and techniques which produce less or no emissions, and the term sustainable means efficient use of resources and production at lower cost [36]. Green innovation assists in handling multiple challenges like emission reduction, cost saving, energy saving, resources efficient utilization, and waste management. To resolve these problems, green innovation can help firms to attain sustainability related objectives [37]. All these benefits through implementing green practices is not easily achieved because firms face barriers in greening the process [38,39].

Green innovation can be classified into a green system, green process, and green product innovation [40,41]. Among various definitions of green innovation, the most elaborated definition is provided by previous studies as “Green innovation is a technique that helps the organizations to meet sustainable growth in a friendly environment through the modified process, system and management” [14,42,43]. Eiadat et al. [44] defined green innovation as a stimulus in manufacturing system that can lead to resource efficiency, embrace the environment system and protect the environment. Although the importance of green innovation has been discussed extensively in the literature [45,46]; however, limited understanding on green innovation barriers is available from the literature, such as Amara et al. [47] argued that understanding the barriers extends the knowledge as to why some firms invest in innovation while others do not. Therefore, it is important to understand why firms do not invest in sustainable practices such as green innovation and how to persuade firms towards green innovation activities.

In developing countries, firms often marred with a lot of obstacles in terms of institutional, resource, and capacity wise [48]. Innovation barriers can be considered separately because of cultural difference [49]. The framework of policies should be aligned with culture, and even similar barriers exist in different national cultures [50]. Therefore, cultural differences give the edge to explore more barriers and on strategies to overcome them offer new insights for future research [51].

### 2.2. Previous Studies Related to Green Innovation

Different researchers defined innovation barriers in multiple ways. According to Oke [52], innovation obstacles stand in the way of innovative activities in the firm. Thus, several barriers to green practices implementation have been identified by researchers in different sectors; in the manufacturing industry, the major obstacles to green innovation adoption include economic resources, attitude and perception, government and customer support. In addition, lack of partnership, insufficient knowledge and information and environmental benefits are also significant barriers to green process innovation [17]. In the study of Giunipero et al. [53] found that green supply chain management is not implemented due to sustainable costs, environmental regulations, and top-level decision misalignment. In the emerging economy of Bangladesh, the lack of resources, consumer demand, and financial concerns arising from short-term limited financial advantage to firms, as well as a lack of government laws, are all significant challenges to implementing green supply chain efforts [54]. The study by Xia, Zhang, Yu and Tu [39] concluded that lack of technologies, lack of infrastructure, insufficient knowledge and resources, and uncertain benefits are key barriers to green technology adoption in Chinese big automotive firms. Jayant and Azhar [55] studied obstacles to the successful implementation of green supply chain management and found 20 barriers; most important barriers are: top management commitment, cost, fear of failure, market, and customer related barriers. Runhaar et al. [56] analyzed recommendations of environmental leaders that support policymaking to going green. They found 26 barriers and major ones were scarce demand for environmental products, high cost, inadequate resources, and customers not interested in paying for environmentally friendly products. Furthermore, Gupta and Barua [57] found lack of resources, financial and human constraints as internal green innovation barriers in manufacturing firms. In another Indian study, the practice of green innovation was examined in the light of barriers, solutions, and policies. Through extensive literature review and opinion of managers, seven main category barriers, 36 subcategory barriers and 20 solutions were identified. In addition, BWM and Fuzzy TOPSIS are used to rank barriers [38]. In a similar study Musaad et al. [58] evaluated six main critical barriers to implementing green innovation in the context of Saudi Arabia. In a recent article, Piyathanavong et al. [59] concluded that an operational sustainability approach has not been implemented rapidly in the Thai economy, due to lack of various sustainability practices in manufacturing firms. da Silva and Gouveia [60] investigated significant obstacles in the way of cleaner production implementation. The main obstacles were economic and technological however, government policies, and customers demand encourage sustainability.

This study contributes to the existing literature on the barriers of green innovation practices in the manufacturing sector by proposing a hybrid decision methodology based on ISM and MICMAC. We extend the literature beyond developed countries because of the lack of empirical studies in developing economies, this studies first find the significant barriers to the implementation of green innovation in the Pakistan manufacturing sector and then created levels to different barriers based on severity.

Our study not only identifies key barriers but also suggest policies to lessen their impact. This way, the practitioners and experts control the environmental challenges and threats to the company. The analysis in the present study would provide a comprehensive and profound insight into the adoption of sustainable green innovative practices in the manufacturing industry. After a thorough review of literature, 20 common barriers to green innovation are identified and are presented in Table 1.

### 2.3. Quest for Green Innovation and Pakistani Manufacturing Sector Perspective

This study focused Pakistan and its manufacturing sector due to its latest and potential growth as well as their impact on environment. By 2030, Pakistan is envisaged to become the 20th biggest economy of the world and will become the 16th largest economy of the world by 2050. The Pakistan manufacturing industry is the main pillar of the economy and it is the second largest sector of the economy contributing 13.5% of the GDP (Government of Pakistan Ministry of Finance, 2019). Moreover, the manufacturing sector is the largest employment generator in the Pakistan economy [92]. According to population density, Pakistan is world’s fifth most populous country having its effect on the pollution. Due to population growth, energy demands have been increased and industries are expanding which has a bad effect on the environment in the shape of noise, air quality, and polluted water. The unplanned system of waste management and chemical usage in manufacturing firms has created environmental problems [93]. Many firms due to non-availability of innovation practices and legislations are threatening the environment that is affecting the quality of life [94]. Recently, Pakistan faces drastic environmental challenges and it ranks seventh among the top 10 most vulnerable country in climate risk index [95]. Although Pakistan manufacturing sectors thriving rapidly but lack the requisite guidance and support for gaining much more insight to drive and improve green performance [96].

### 2.4. Critical Review of Green Innovation Studies and Study Framework

Although some authors have studied different aspects of green practices. Majority of the work related to conceptual framework basis or structural basis [97,98]. Recent studies of Gupta and Barua [38]; Xia, Zhang, Yu, and Tu [39]; and Musaad, Sultan, Zhuo, Musaad, Otaibi, Siyal, Hashmi, and Shah [58] have investigated green innovation barriers however, very few studies exist in manufacturing industry [77]. Furthermore, the studies conducted on green innovation have focused on few factors at a time and are often limited to finding the effect of one barrier on other. There is no such study to first find the interrelationship among barriers. This study aims to first find the hierarchal structure and dependence relationship among identified barriers in developing country’s manufacturing sector. Organizations and decision makers need to have a framework through which they can identify barriers to green innovation in general. We propose a novel framework for analyzing the barriers to green innovation adoption that we explored. This framework has a defined as well as a reliable nomenclature [99]. The following are the major steps in the proposed research framework:

(a) Procedures pertaining to the current framework, such as selection of common barriers, the collection of relevant literature, and the applicability of the research techniques are all connected to this goal.
(b) Every structural technique is supported by literature review, which has been approved by professionals. The barriers identified through extensive literature survey and then filtered with specific data.
(c) This step entails finding the link between the identified barriers and developing hierarchical levels for the barriers to green innovation adoption. The ISM MICMAC technique is utilized for this purpose.
(d) The outcomes of this research will help managers and specialists. This framework also aids managers in (a) picking suitable barriers to green innovation implementation, (b) recognizing barriers’ linkages, and (c) establishing hierarchical levels of green innovation implementation challenges.

### 2.5. Research Gap

Green innovation adoption is merely an economic tool that produce new products at lower manufacturing cost [100]. Firms may accomplish a competitive advantage by engaging in green innovation strategically but, they experience a lot of obstacles in pursuing green innovation and introducing new product or process in the market, therefore, studying factors affecting green innovation process should grasp crucial insights for managers to overcome green innovation barriers [20]. There is a misconception that every country experiences the same innovation and environmental issues. The scale of the barrier’s importance may vary due to specific country scenarios. The majority of previous studies related to advanced economies and very few, centered low-income economies. Although several studies researched on barriers in small and medium enterprises (SME, s) e.g., [23,25,38,75,79]; however, studies on manufacturing sector are not adequate to deal with it, and hence it requires further exploration [17]. Developing nations, like Pakistan observes paucity of rules and regulation for adopting green initiatives and sustainable practices. The identification and overcoming barriers to green innovation in the manufacturing sector can make the environment safe and healthy for all stakeholders.

## 3. Methodology

**Figure 1:**
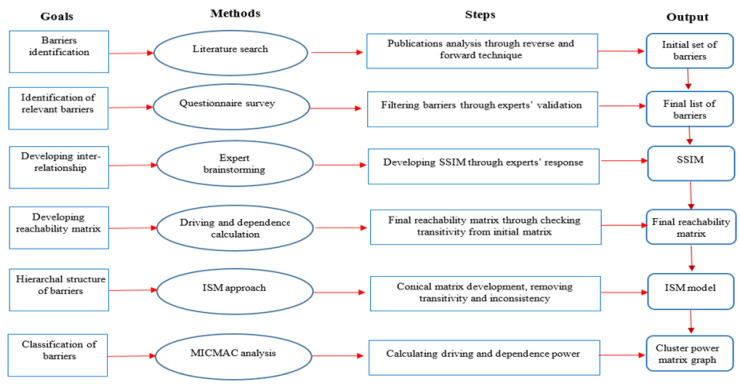
Detailed approach/methodology towards identifying green innovation barriers.

To investigate the barriers hindering the potential benefits of green innovation in manufacturing sectors, the current study approaches different steps to identify green innovation barrier’s structure. The detailed approach is shown in Figure 1.

### 3.1. Identifying Initial Set of Barriers to Green Innovation (Literature Search—Step 1)

To ascertain the barriers of green innovation, a systematic review of literature is essential regarding this concerning topic. A detailed literature survey was conducted using different keywords or a combination—e.g., “Green, sustainable, Green innovation, Barriers, Hampers, Obstacles, manufacturing, and green innovation in Pakistan”, “Innovation barriers in the manufacturing sector, green initiatives in the manufacturing sector, Green and sustainable adoption barriers in the manufacturing sector, Green innovation in manufacturing sectors and developing countries”. The keywords were searched in famous search engines and databases such as Google Scholar, Scopus, Springer, EBSCO, Emerald, Taylor and Francis, Science Direct, and Wiley Online. The inclusion/exclusion criteria are mandatory for systematic review studies [101]. Inclusion criteria are set as follows: (a) Peer review published journals in English; (b) studies regarding the systematic review and innovation barriers in the manufacturing sector; (c) studies involving multiple cases of several countries as an example, such as [102] performed an empirical analysis of 14 industrial sectors in OECD countries. On the other hand, exclusion criteria set as follows: (a) studies mainly focus on high quantitative methods and analysis avoiding qualitative aspects; (b) studies on multiple case studies in different sectors other than manufacturing; (c) studies comprises ambiguous methodologies representation; (d) studies proposes methodologies, tools, methods on green innovation. Finally, 300 papers were identified using the mentioned databases. To screen out papers, a methodology by Zhu, Groening, and Sarkis backward and forward technique were employed. At last, 40 articles related to our study were considered. Through expert’s views and detailed literature help, a total of 20 impediments were finalized in first stage.

### 3.2. Identification of Relevant Barriers through Fuzzy Delphi Method (FDM) (Questionnaire Survey—Step 2)

Due to complexity of the green innovation technology, the selection of barriers in green products can be viewed as a multi-phase decision process that is viewed by expert’s team, government, and industry. Fuzzy Delphi method is heuristic technique to solve the problems on the basis of expert’s opinion. There are few rounds of discussion in which experts submit their responses to the facilitator. The facilitator makes summary of all responses, provide number scale, and presents in front of experts to reconsider the respective responses again. This way encourages the experts to change their answers which mitigate the number of variables affecting the system, and investigate the most relevant variables.

Data were obtained from the five firms of Pakistani manufacturing industry that are responsible for pollution, including cement, textile, steel, oil & gas, and leather. Our study takes empirical observation from the large-scale Pakistani manufacturing companies as previous studies investigated that the size of organizations affects the volume of environmental performance [103]. The manufacturing experts were contacted through e-mails, phone calls, and personal visits in the office. In the preliminary stage, 16 professionals from the manufacturing area were called and briefed about green innovation barriers. Then, only seven experts and three academicians showed their interest in the study. Eventually, a team of six experts intended to participate, comprising one environmental expert, one manufacturing specialist, one R&D manager, one quality manager, one project manager, one technological specialist, and one operation manager. The selected members were highly professional and trained in their field. The demographics bio data is presented in Table 2.

This study uses two phases procedure, the first stage utilizes the collection of responses in the shape of questionnaire in which triangular fuzzy numbers are given to highlights the severity of green innovations barriers. The next stage is calculating the fuzzy weight Wwk of green innovation obstacles into a single value Vk; here, Wwk shows the total fuzzy numbers (TPNs), achieved with support of three values of fuzzy numbers (geometric mean (GM), maximum value, and minimum value). Using the center of gravity rule, we get

Vk = (Maximum value, Minimum value, GM)/3.

Here, Vk shows the initial value set for filtering the most suitable green innovation barriers for analysis. After two iterations of FDM in a preliminary list of 20 green innovation barriers, the experts’ consensus reached on the ground to retain 18 green innovation barriers for further evaluation. After briefing and analyzing the objectives of the research with the group panel, the ISM technique was selected. The variables hindering the green innovation were identified with a literature survey and then, filtered more related barrier through expert opinions.

The process of investigation and filtration of green innovation barriers are shown in Table 1.

The next level is forming a contextual relationship between variables (barriers) with expert guidance. For this purpose, expert panel sessions were organized to find out barriers causing the successful implementation of green innovation in the manufacturing sector and their contextual relationships. The experts were asked to provide answers and to show contextual interactions.

Furthermore, a questionnaire was prepared based on expert’s opinions in the light of significance and relevancy of barriers. The pairwise interactions among factors were established with expert’s proper guidance for making structural self-interactions matrix (SSIM).

### 3.3. Application of the Interpretive Structural Modeling (ISM) Method (Steps 3–5)

Interpretive structural modeling (ISM) is a well-known technique widely used method in social sciences for addressing interactions among systems and elements [104]. To formulate the relationship, the ISM technique approaches expert’s opinion and information to interrelate in a well-designed multi structural way.

The ISM methodology is composed of the following steps [105].

1. Those factors affecting the process are listed. This research identifies the obstacles impeding the successful adoption of green innovation in manufacturing sectors are considered as factors.
2. Contextual linkage between the investigated factors is formed.
3. Pairwise relationships among factors are developed through the formulation of structural self-interaction matrix (SSIM).
4. The reachability matrix is established to check the transitivity. The transitivity rule assumes that A has relationship with B and B has a relationship with C then, A has an obvious relationship with C.
5. Final reachability is constructed through applying transitivity rule which is divided into different parts.
6. A directed graph is drawn based on relationships for the final reachability matrix, and the transitive links are removed.
7. The final digraph is changed into an ISM by converting element nodes with statements.
8. To ensure valid results, the theoretical interpretive structural model is retested in case of inconsistency and adjustments must be made.

**Figure 2:**
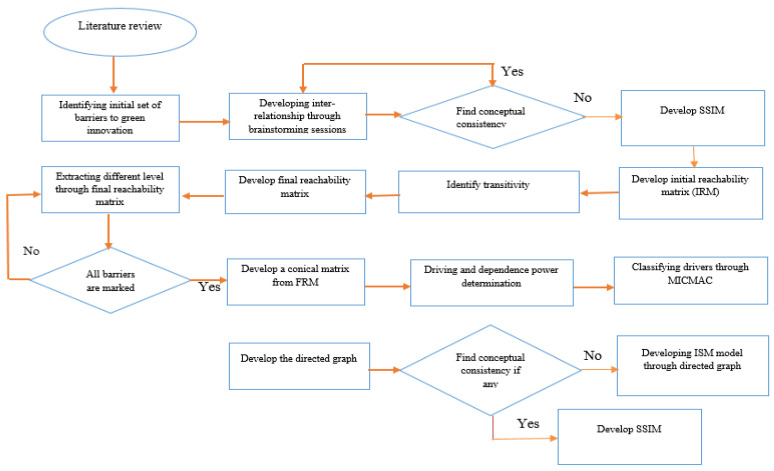
Application of ISM approach to develop an ISM model of green innovation barriers.

Steps involved in ISM modeling are shown in Figure 2.

The contextual relationship of green innovation barriers were obtained from participants. Table 3 presents’ different relationships symbols which are explained below by the cell number:

1. Cell 1–2: The relationship between the Lack of top management commitment and lack of training and seminars related to green innovation. All the participants stated that: “due to lack of top management support firms cannot organize training and seminars related to green innovation. Top management plays key role in environmental training held in organizations”.
2. Cell 1–3: The relationship between the lack of top management commitment and insufficient human resources for green innovation. The participants stated that non-seriousness of environmental leadership leads to lack of human personnel to adopt green practices. Top management plays key role for developing strategies and policies, hiring and training of related personnel. Similarly, same way the remaining barriers relationships are constructed using the symbols.

#### 3.3.1. SSIM Construction

The expert’s panel of the manufacturing sector and practitioners analyzed the relationships between variables to construct a structure using a self-interaction method. These relationships were shaped in pairs so, experts are asked to develop a contextual relationship between factors (barriers) [106].

SSIM was established according to contextual interactions and below four signs were employed.

V Barrier i will help to influence barrier j,
A Barrier j will help to influence barriers i,
X Barriers i and j influence each and others,
O Barriers i and j are not related.

The SSIM for barriers to green innovation implementation was developed based on mentioned symbols and expert opinions in Table 3.

#### 3.3.2. Reachability Matrix Formation (IRM)

For constructing reachability matrix, the SSIM was converted into binary values in the light of transitivity rule. The letters O, A, X, and V in entries (j,i) or (i,j) delineates interactions connecting the barrier number ‘j’ in the column of the cell with the barrier number ‘i’ in the line. The detail briefings are explained below and results are provided in Table 4.

(1) For each cell (i,j) containing “V” we insert 1 and 0 for (j,i) cell.
(2) For each cell (i,j) containing “A” we insert 0 and 1 for (j,i) cell.
(3) For both cells (i,j) and (j,i) containing “X” we insert 1.
(4) For both cells (i,j) and (j,i) containing “O” we insert 0.

#### 3.3.3. Final Reachability Matrix

Following ISM methodology, the final reachability matrix was developed by removing the transitivity from the IRM, as depicted in the Table 5. The final reachability used to know the different levels of hierarchy.

#### 3.3.4. Level Partitions and ISM Model Formulation

After the final reachability matrix (FRM) process, the next step is to prepare reachability, intersection, and antecedent sets. The reachability set consists itself and other barriers it leads to; however, the antecedent comprises itself and other elements it affects. In intersection set, we take common values from both reachability and antecedent sets following the same procedure for all elements. The factors (barriers) in which reachability and antecedents found the same are assigned (level 1)—e.g., fear of failure about green innovation. Then, the assigned barriers levels are eliminated to reduce redundancy. The process is repeated for remaining variables until each variable assigned at least one level. The level partitions for all barriers in green innovation adoption are presented in Table A1, Table A2, Table A3, Table A4 and Table A5 (see Appendix A). The procedure is thoroughly applied to all barriers and includes five levels in total.

**Figure 3:**
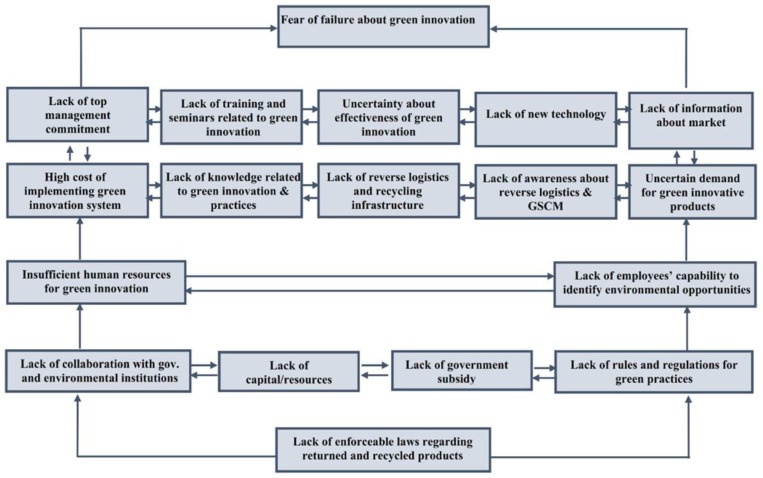
ISM based model and hierarchal level of barriers affecting green innovation implementation in the manufacturing industry of Pakistan.

After partitioning barriers, we formulated a structural hierarchy model of all barriers as presented in Table 6. The partition process helps to construct each level of hierarchy which shows their degree of importance. These finding in the ISM model are formulated in Figure 3. In the hierarchy model, top level 5 barrier; fear of failure about green innovation (B7) is considered weak driving because its role in affecting other barriers is narrow. The barrier at the bottom level 1—e.g., lacks enforceable laws regarding returned goods and recycled products (B17) are the most critical barrier. These two levels, level 5 and level 4 are the most influential barriers affecting the effective innovation process. The barriers lying in the middle of the model are level 2 and level 3. These levels are playing mediating and linkage role between the top and bottom levels. The ISM model is presented in Figure 3.

### 3.4. MICMAC Analysis

**Figure 4:**
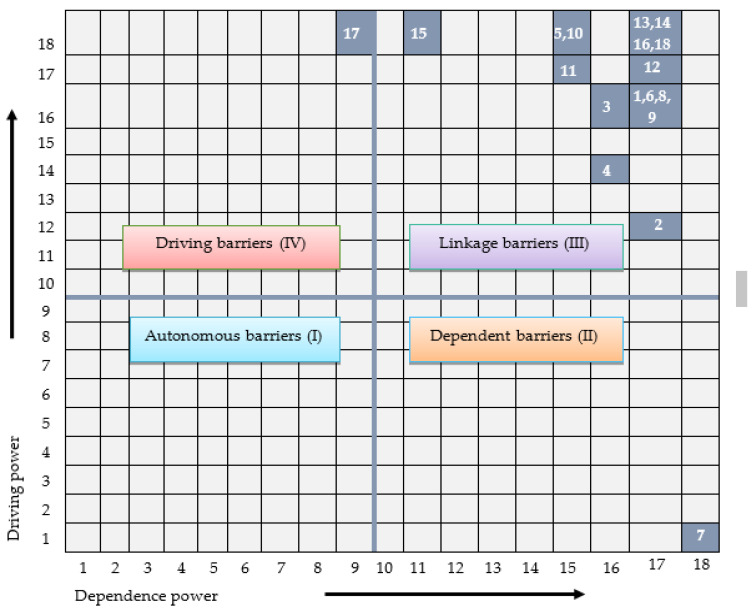
MICMAC analysis of the barriers for the green innovation adoption.

In this study, MICMAC is incorporated to identify critical factors based on dependence and driving force. From the reachability matrix, the dependence and driving power of each of the green innovation factor was calculated. The dependence power is the sum of all values in the column leading to that element (Table 5). The driving power is extracted by adding the entire values of that element in a row. Here, the dependence power factor articulates that other factors affect that factor while the driving force depicts the factors it is driving. The identified barriers were classified into four quadrants considering the driving and dependence power derived from MICMAC analysis as presented in Figure 4. The MICMAC analysis is explained below:

Autonomous: These are the barriers having weak dependence and weak driving power. In this study, no element belongs to this category.

Dependent: These are the factors with a weak driving force but a strong dependence power. This category comprises one barrier at the top of the hierarchy in the model which is ‘fear of failure about green innovation (B7)’.

Linkage: These are the barriers with strong driving power as well as dependence power. There are total 16 barriers identified as mediating variables in the middle consisting. Lack of top management commitment (B1), Lack of training and seminars related to green innovation (B2), Insufficient human resources for green innovation (B3), Lack of employees’ capability to identify environmental opportunities (B4), Lack of collaboration with government and environmental institutions (B5), Uncertainty about the effectiveness of green innovation (B6), Lack of new technology (B8), Lack of information about market (B9), Lack of capital/resources (B10), Lack of government subsidy (B11), High cost of implementing green innovation system (B12), Lack of knowledge related to green innovation and practices (B13), Lack of reverse logistics and recycling infrastructure (B14), Lack of rules and regulations for green practices (B15), Lack of awareness about reverse logistics and green SCM (B16), and Uncertain demand for green innovative products (B18).

Driving or independence: These are the barriers with a strong driving power but a weak dependence and are found at the bottom of the model. In this portion, only one barrier ‘Lack of enforceable laws regarding returned and recycled products (B17)’. This barrier creates a foundation for other barriers and so-called root cause because various factors are affected through it. The practitioners and manufacturing specialists should carefully evaluate to eliminate this factor.

## 4. Results and Discussion

We investigated green innovation barriers in developing economies. After studying significant barriers of green innovation in literature, a total of 20 barriers were selected initially and then 18 green innovation barriers finalized for the study. All impeding factors suitable for the Pakistan environment were considered with the help of professional experts. A layout was prepared to mitigate or lessen their strength

To reduce mistakes, panel experts checked the interpretive structural model and the aforementioned results of the MICMAC technique. Experts were satisfied to accommodate the possible barriers relating to the present scenario. The results are displayed in structural discussion form under the levels of barriers identified from the ISM model.

This study found ‘lack of enforceable laws regarding returned and recycled products’ B17 as a major barrier in case analysis. This factor is considered a major hurdle for the country’s manufacturing unit. In less developed countries, regulations are barely entrenched and as a result, stakeholders only seek economic gains without pursuing social objectives [107]. However, legislation awareness in society to adopt sustainable practices is essential [108]. Lack of enforceable laws regarding returned and recycled products not only causing problems in the manufacturing sector but in various sectors as well. Like Waqas et al. [89], Abdulrahman et al. [90] identified lack of enforcement of environmental laws as a critical obstacle in implementing green practices—e.g., reverse logistics in the manufacturing sector in developing economies. Furthermore, the system of reverse logistics to produce environmentally friendly products is not applicable due to weak environmental regulations. Furthermore, Azeem et al. [109] documented that poor implementation of laws and legislations is a major issue in the adoption of green construction in the Pakistan construction sector. The author added, the government should not only play a role in the creation of policies but effectively implement rules and regulations through regular monitoring and assessment. The presence of this barrier in green innovation is creating a terrible situation for the manufacturing sector. This barrier is deeply linked with another hierarchy of barriers and constitute level 4.

Accordingly, at level, lack of enforceable laws regarding returned goods and recycled products help achieve in ‘lack of collaboration with government and environmental institutions (B5); lack of rules and regulations for green practices (B15); lack of capital/resources (B10); and lack of government subsidy (B11)’ accordingly. There is a problem in finding external partners with working and cooperating on green innovation initiatives in firms. The reason is that the innovation payback period is long and costly, and financing is difficult [110]. Another major barrier that impede corporate green innovation practices is lack of rules in the manufacturing sector. Oelze [83] concluded that lack of rules and regulations is a significant barrier to implement sustainable supply chain management. In similar studies, lack of rules and regulations has been acknowledged as an important critical barrier in the implementation of green supply chain management and reverse logistics [53,55,111]. The lack of capital/resources and lack of government subsidy is deterring firms from green innovation practices. Furthermore, weak financial assistance and a lack of incentive system makes the innovation process more difficult to implement [79].

At this Level 3, insufficient human resources for green innovation (B3) and lack of ability of employees to identify environmental opportunities (B4) are identified. These are human related barriers affecting the implementation process of green innovation in case companies. Owing to the lack of skilled personnel, top management does not show a keen interest in innovative activities [81], because of poor knowledge and capability to identify environmental benefits. Furthermore, Galia and Legros [112] and Baldwin and Lin [113] explored a lack of skilled personnel as a significant impediment in adopting innovation practices in manufacturing firms.

Level 3 barriers (B3) and (B4) leads to lack of top management commitment (B1); lack of awareness about reverse logistics and green SCM (B16); lack of reverse logistics and recycling infrastructure(B14); lack of knowledge related to green innovation and practices (B13); high cost of implementing green innovation system (B12); lack of information about market (B9); lack of new technology (B8); uncertainty about the effectiveness of green innovation (B6); lack of training and seminars related to green innovation (B2); uncertain demand for green innovative products (B18) following the model, these barriers exist in level 3. This study identified ten obstacles affecting the green innovations process and among them ‘lack of top management commitment’ is the most damaging barrier of initiating green practices in companies [114]. The top management is not committed because of poor knowledge of employees and other internal firm factors [61]. However, green practices cannot be implemented without top management support [64]. This study identifies that top leadership is not serious in implementing green practices. The manufacturing of green products incurs an additional cost that is critical barriers in the green supply chain management implementation phase [115] and in a developing country—e.g., Pakistan—firms often face resource constraints so many firms do not actively involved in green practices. Therefore, this issue leads further corresponding factors; lack of training and knowledge related to green innovation and practices [63] that means manufacturing firms do not embark innovative activities and hence, firms may hesitate to acquire a new plant or machinery [116]. Our study also found that green innovation is not incorporated due to insufficient information about the market. Madrid-Guijarro et al. [77] mentioned that limited information about market block firms from the next level of product or process innovation as they feel uncertain in the market. The market and knowledge related obstacles in green innovation adoption have so far ignored [17]. Furthermore, if sustainable products do not bring real benefits to the company then, manufacturing firms do not adopt green practices as green products are expensive so, the demand for green products remains uncertain [17,32]. In emerging economies, the market faces low awareness about green practices in the supply chain while, these emerging markets pose serious threats to the environment [117]. Furthermore, the implementation of reverse logistics and green supply chain management has been a challenging factor for developing economies such, as Pakistan [59]. However, the implementation of green supply chain management can restrain environmental dangers [118]. At last, level 5, all barriers culminate into fear of failure about green innovation (B7) [74].

## 5. Policy Implications to Overcome Green Innovations Barriers

This section addresses possible solutions to barriers encountered in the effective process of incorporating green innovation in Pakistan manufacturing companies. Below, policies and possible solutions to mitigate the effect of barriers are provided.

### 5.1. The Solution to the First Level Barrier

Lack of enforceable laws regarding returned goods and recycled products (B17) is a macro and national level issue and cannot be solved by the manufacturing sector alone. The success of green practices and its implantations is largely dependent on government policies [119]. Ortolano et al. [120] investigated that environmental regulation enforcement is the main motivator in cleaner production processes in Pakistan leather and textile industry that can stimulate green innovation. Therefore, public policies have a significant role to play; they must raise firm’s awareness, provide the necessary information [14]. Pakistan’s environmental protection agency (PAK-EPA) can mitigate this barrier by enforcing environmentally friendly policies.

### 5.2. The Solution to Second Level Barriers

This level comprises B5, B10, B11, and B15. The government can mitigate second level barriers by providing debt financing, equity financing, subsidies, and launching public private partnership projects. The government and policymakers should substantially promote policy frame and financial support to sustainable development [59]. A carbon tax can motivate firms to innovate green products which can reduce green carbon emissions. Financial problems can be controlled through private and public and state investment banks—e.g., infrastructure investment [121]. Furthermore, the government can relieve financially through concession on import export duty and tax on environmental technologies. The environmental infringements by firms should be panelized according to rules.

### 5.3. The Solution to Third Level Barriers

This level comprises B3 and B4. Pakistan is a developing country that is not equipped to deal with current environmental issues. The manufacturing industry should attract employees, train personnel who can translate into green products manufacturing [17]. Therefore, policymakers need to develop necessary skills, knowledge, technological training that can be effective [122,123].

### 5.4. The Solution to Fourth Level Barriers

This level consists of 10 barriers B1, B16, B14, B13, B12, B9, B8, B6, B2, and B18. The lack of top management commitment (B1) is a significant barrier in green concepts adoption in Pakistan manufacturing industry. The top management commitment is an internal force that supports proactive sustainability behaviors as well as the successful implementation of sustainability initiatives [26,53,124]. In this respect, top management should not only determine to set innovative goals and long-term strategies, but also motivate and train employees towards green initiatives. The uncertainty in demand for green innovative products can be mitigated through communicating the benefits of environmentally friendly practices to all stakeholders. Such practices involve reusability, fixing, durability, and recycling of products used in the manufacturing sector. Policymakers and governments should study market related barriers properly [58]. The lack of reverse logistics practices and green supply chain management issues can be resolved through government policies, greening the logistics and encouraging the manufacturers by providing a certificate scheme to support sustainable agenda. The government should provide bonuses and incentives to environmentally friendly logistics and impose heavy penalties on high polluted supply chain [64]. The high innovation cost barrier can be addressed through government support in the shape of an incentive package and subsidizing green products [125].

Developing green innovation software for manufacturers where manufacturing firms can access to latest innovation information, technologies, and issues related to green products [126], that tends to lessen the barrier ‘lack of information’. The technological barrier can be removed through investment in green research and development to design green products [100]. The financiers of clean technologies and policy makers’ roles in designing effective policies and frameworks are to reduce uncertain investments into calculable risk and return can decrease the uncertainty in projects [127]. More, organizing training and seminars on green innovation can bring changes in their innovative decisions as these provide opportunities and expertise to bring new ideas in an environmentally conscious manufacturing (ECM) process. Such education and seminars will boost organizations sustainability performance [65].

### 5.5. The Solution to Fifth Level Barriers

Finally, this level has only one barrier ‘fear of failure B7’ which can be managed through adopting effective strategies of return on investment such as recovery, by products and reselling to decrease wastage of material. Furthermore, investing in green practices can increase profitability; therefore, companies should consider green innovation as a prime activity and pay more for green innovation.

Additionally, green innovation concepts should be adopted immediately to cope with recent environmental issues such as global warming, energy crisis, and water shortages. Resources allocation and efficient utilization should be made and implementing reforms in the manufacturing sector.

### 5.6. Comparison with Other Countries

In addition, the top five barriers to green innovation in the manufacturing sector of Pakistan were compared with those identified in earlier studies in different countries, as shown in Table 7. The results of the current study are interesting, because lack of enforceable laws regarding returned and recycled products emerged as the top barrier. However, as Table 7 indicates, it does not feature among the top five barriers of other countries. Recent study of Waqas et al. [128] mentioned that lack of enforceable laws on product return is a major barrier in reverse logistics in Pakistan manufacturing sector. Earlier studies identified the lack of financial support [57], implementation of environmental policies [58], additional cost due to green innovation as top ranked barriers to green innovation practices. This study found that lack of enforceable laws regarding returned and recycled products further leads to a lack of rules and regulations for green practices. Lack of rules and regulations have ranked top level barriers in the literature; like a study based in Saudi Arabia ranked lack of implementation of environmental policies as a top-level barrier to green innovation practices. However, while there could be different reasons for a lack of rules and regulation, in this study, it was driven by a lack of enforceable laws regarding returned and recycled products. It will be more interesting to know the reasons for the lack of rules and regulation to green innovation adoption in different countries with different contexts.

Lack of collaboration with government and environmental institutions was the third key barrier to green innovation adoption. It also does not feature in the top five barriers highlighted in earlier studies. A lack of capital/resources and lack of government subsidy were ranked fourth and fifth, respectively in this study. Earlier studies highlighted financial barriers. For example, a study in Hong Kong ranked additional costs due to green requirements first while, a study on the manufacturing sector of India stated lack of financial support as top ranked barriers to green innovation.

## 6. Conclusions, Managerial Implications, and Future Research

Green innovation has gained huge cognizance around the globe. Manufacturers, producers, as well as consumers are finding out ways to extenuate the impact of industrialization on the environment. The production of green products, reverse logistics, and adoption of green practices are still at the nascent stage in developing economies like Pakistan. The manufacturing industry—being bigger in size—is considered the backbone of the economy, which is facing significant obstacles in the adoption of green concepts. Green innovation is the best solution to this Armageddon, but successful implementation is still a challenging task for developing countries. There are scant studies relating to barriers of green innovation adoption in developing economies. For this reason, the present study investigated key barriers; establish their contextual relationship with hierarchal levels from vast literature and expert’s assistance. The most dominant factors found lack of enforceable laws regarding returned goods and recycled products and lack of government support while fear of failure about green innovation is the least critical barrier in the study. Our study addresses policies to cure the pathetic situation of the environment which can spark stakeholders to become more environmentally conscious.

After long discussion and analysis applying the ISM model and MICMAC methods, we reach the following managerial implications. The initial stage is to introduce green philosophies in manufacturing firms that will reap sustainable development. Therefore, the government should focus on green development in the manufacturing industry and formulate systematic policies to conjure the development of economy, energy, and environment. The environmental policy should work with the practices of the manufacturing industry by improving the system along with environmental information disclosure and environmental protection. The next stage is the Kaizen concept towards the environment. The continuous effort to eliminate waste from all manufacturing processes, logistics, or activities is required.

The results displayed a true picture of the Pakistani context for green innovation adoption. The combination of the ISM model and MICMAC does not explain why one barrier is affecting or being influenced by other barriers. Thus, to exemplify this problem for logical interactions among barriers we highly recommend using total interpretive structural modeling (TISM) for future work. Our study revealed another gap for potential future research work that can be utilized in future work by applying structural equation modeling (SEM) to check and verify our results. Furthermore, the results can be verified through other techniques like analytical hierarchy process (AHP), decision-making trial and evaluation laboratory (DEMATEL), analytic network process (ANP), and the fuzzy approach.

## Figures and Tables

**Table 1:** Summary of key barriers of green innovation implementation.

S#	Impediment to Green Innovation	Descriptions	Sources
1	Lack of top management commitment	Lack of top management in green practices is considered a major impediment but the success of sustainable practices depends on top management commitment.	[38,61,62,63,64]
2	Lack of training and seminars related to green innovation	Lack of training and programs may slow the pace of green innovation as technical competencies play a vital role.	[58,63,65,66]
3	Insufficient human resources for green practices	Insufficient work force is a big challenge in the success of green practices.	[67,68]
4	Lack of employees’ capability to identify environmental opportunities	Untrained employees are unable to find the environmental opportunities.	[69,70]
5	Lack of collaboration with government and environmental institutions	Weak partnership between firm and government hinders the effective green process however, their collaboration is essential for green innovation adoption.	[17,38]
6	Uncertainty about the effectiveness of green innovation	Green innovation is risky in terms of uncertain returns perspectives.	[69,71,72,73]
7	Fear of failure about green innovation	This factor is critical to adopt green innovation.	[74]
8	Lack of new technology	The degree of technological information for understanding green innovation is high level but firm’s embraces low information face sustainability constraints.	[63,75,76]
9	Lack of information about the market	Green innovations requisite certain information to adopt eco practices successfully in the market.	[17,24,77]
10	Lack of capital/resources	Poor financial resources preclude organizations from implementing environmental plans.	[23,78]
11	Lack of government subsidy	Government incentives and subsidies play a key role to motivate green manufacturing.	[25,79]
12	The high cost of implementing green innovation system	High costs associated with manufacturing green products make it difficult to adopt green practices.	[79,80]
13	Lack of knowledge related to green innovation and practices	Lack of knowledge blocks the innovation process.	[63,65,69,72,81]
14	Lack of reverse logistics and recycling infrastructure	Green innovation requires sophisticated technology to absorb, reuse/recycle wastes during manufacturing.	[82]
15	Lack of rules and regulations for green practices	Unclear rules and regulations do not bind firms to follow environmental regulations.	[56,83,84]
16	Lack of awareness about reverse logistics and green SCM	Within companies there’s less conscious of recycling and disposing of products that affect the sustainability process.	[55,85,86,87,88,89]
17	Lack of enforceable laws regarding returned and recycled products	Organizations are often demotivated due to poor enforcement of environmental laws and hence, few firms take advantage of it.	[84,89,90]
18	Uncertain demand for green innovative products	Green product demand is uncertain due to high cost and uncertain returns, so firms hesitate to produce green products.	[17,81,91],
19	Attitude and perception *	Some organizations wrong perceptions about green innovation that it cannot help to improve their business.	[17]
20	Business practices * barriers	Business practices such as lean, sig sigma, and ISO and innovative technologies help organizations to be competitive and failing to adopt new initiatives restricts the business.	[17]

* Green innovation barriers deleted through FDM.

**Table 2:** Demographics characteristics of experts.

S.No	Position	Gender	Age	Years of Experience	Education Level
1	Environmental expert	Male	65	15	Ph.D.
2	Manufacturing specialist	Male	60	15	Ph.D.
3	R&D manager	Female	64	11	Ph.D.
4	Quality manager	Male	58	14	Master
5	Project manager	Male	54	12	Master
6	Technological specialist	Female	62	10	Ph.D.

**Table 3:** SSIM.

No.	Variables	1	2	3	4	5	6	7	8	9	10	11	12	13	14	15	16	17	18
1	Lack of top management commitment		V	V	O	V	A	V	X	X	X	A	A	V	V	A	V	O	O
2	Lack of training and seminars related to green innovation			A	V	O	V	V	X	V	A	O	A	V	O	O	V	A	O
3	Insufficient human resources for green innovation				V	V	O	V	O	V	A	A	A	V	V	O	V	O	O
4	Lack of employees’ capability to identify environmental opportunities					A	V	V	X	V	A	O	V	A	A	O	A	O	V
5	Lack of collaboration with government and environmental institutions						V	V	V	V	A	V	V	V	V	V	V	O	O
6	Uncertainty about effectiveness of green innovation							V	O	A	A	A	O	A	A	O	A	O	A
7	Fear of failure about green innovation								A	A	O	O	A	A	O	O	O	O	O
8	Lack of new technology									X	A	A	V	A	V	O	O	O	O
9	Lack of information about market										A	A	V	X	O	O	V	O	O
10	Lack of capital/resources											O	X	V	V	O	V	O	A
11	Lack of government subsidy												V	O	V	A	V	O	O
12	High cost of implementing green innovation system													X	A	O	A	O	O
13	Lack of knowledge related to green innovation and practices														V	A	X	A	O
14	Lack of reverse logistics and recycling infrastructure															A	A	A	O
15	Lack of rules and regulations for green practices																V	V	O
16	Lack of awareness about reverse logistics and green SCM																	A	V
17	Lack of enforceable laws regarding returned and recycled products																		O
18	Uncertain demand for green innovative products																		

**Table 4:** IRM.

No.	1	2	3	4	5	6	7	8	9	10	11	12	13	14	15	16	17	18
1	1	1	1	0	1	0	1	1	1	1	0	0	1	1	0	1	0	0
2	0	1	0	1	0	1	1	1	1	0	0	0	1	0	0	1	0	0
3	0	1	1	1	1	0	1	0	1	0	0	0	1	1	0	1	0	0
4	0	0	0	1	0	1	1	1	1	0	0	1	0	0	0	0	0	1
5	0	0	0	1	1	1	1	1	1	0	1	1	1	1	1	1	0	0
6	1	0	0	0	0	1	1	0	0	0	0	0	0	0	0	0	0	0
7	0	0	0	0	0	0	1	0	0	0	0	0	0	0	0	0	0	0
8	1	1	0	1	0	0	1	1	1	0	0	1	0	1	0	0	0	0
9	1	0	0	0	0	1	1	1	1	0	0	1	1	0	0	1	0	0
10	1	1	1	1	1	1	0	1	1	1	0	1	1	1	0	1	0	0
11	1	0	1	0	0	1	0	1	1	0	1	1	0	1	0	1	0	0
12	1	1	1	0	0	0	1	0	0	1	0	1	1	0	0	0	0	0
13	0	0	0	1	0	1	1	1	1	0	0	1	1	1	0	1	0	0
14	0	0	0	1	0	1	0	0	0	0	0	1	0	1	0	0	0	1
15	1	0	0	0	0	0	0	0	0	0	1	0	1	1	1	1	1	0
16	0	0	0	1	0	1	0	0	0	0	0	1	1	1	0	1	0	1
17	0	1	0	0	0	0	0	0	0	0	0	0	1	1	0	1	1	0
18	0	0	0	0	0	1	0	0	0	1	0	0	0	0	0	0	0	1

**Table 5:** FRM.

No.	1	2	3	4	5	6	7	8	9	10	11	12	13	14	15	16	17	18	Driving Power
1	1	1	1	1 *	1	1 *	1	1	1	1	1 *	1 *	1	1	0	1	0	1 *	16
2	1 *	1	0	1	0	1	1	1	1	0	0	1 *	1	1 *	0	1	0	1	12
3	1 *	1	1	1	1	1 *	1	1 *	1	0	1 *	1 *	1	1	1 *	1	0	1 *	16
4	1 *	1 *	1 *	1	0	1	1	1	1	1 *	0	1	1 *	1 *	0	1 *	0	1	14
5	1 *	1 *	1 *	1	1	1	1	1	1	1 *	1	1	1	1	1	1	1 *	1 *	18
6	1	1 *	1 *	1 *	1 *	1	1	1 *	1 *	1 *	1 *	1 *	1 *	1 *	0	1 *	0	1 *	16
7	0	0	0	0	0	0	1	0	0	0	0	0	0	0	0	0	0	0	1
8	1	1	1 *	1	1 *	1 *	1	1	1	1 *	1 *	1	1 *	1	0	1 *	0	1 *	16
9	1	1 *	1 *	1 *	1 *	1	1	1	1	1 *	1 *	1	1	1 *	0	1	0	1 *	16
10	1	1	1	1	1	1	1 *	1	1	1	1 *	1	1	1	1 *	1	1 *	1 *	18
11	1	1 *	1	1 *	1 *	1	1 *	1	1	1 *	1	1	1 *	1	1 *	1	0	1 *	17
12	1	1	1	0	1 *	1 *	1	1 *	1 *	1	1 *	1	1	1 *	1 *	1 *	1 *	1 *	17
13	1 *	1 *	1 *	1	1 *	1	1	1	1	1 *	1 *	1	1	1	1 *	1	1 *	1 *	18
14	1 *	1 *	1 *	1	1 *	1	1 *	1 *	1 *	1 *	1 *	1	1 *	1	1 *	1 *	1 *	1	18
15	1	1 *	1 *	1 *	1 *	1 *	1 *	1 *	1 *	1 *	1	1 *	1	1	1	1	1	1 *	18
16	1 *	1 *	1 *	1	1 *	1	1 *	1 *	1 *	1 *	1 *	1	1	1	1	1	1	1	18
17	1 *	1	1 *	1 *	1 *	1 *	1 *	1 *	1 *	1 *	1 *	1 *	1	1	1 *	1	1	1 *	18
18	1 *	1 *	1 *	1 *	1 *	1	1 *	1 *	1 *	1	1 *	1 *	1 *	1 *	1 *	1 *	1 *	1	18
Dep. power	17	17	16	16	15	17	18	17	17	15	15	17	17	17	11	17	9	17	285

Note: * indicates transitive links.

**Table 6:** Hierarchal levels and barriers at each level.

1	Fear of failure about green innovation (B7)	Fifth
2	Uncertain demand for green innovative products (B18)	Fourth
	Lack of awareness about reverse logistics and green SCM (B16)	
	Lack of reverse logistics and recycling infrastructure (B14)	
	Lack of knowledge related to green innovation and practices (B15)	
	The high cost of implementing green innovation system (B12)	
	Lack of information about the market (B9)	
	Lack of new technology (B8)	
	Uncertainty about the effectiveness of green innovation (B6)	
	Lack of training seminars and connected to green innovation (B2)	
	Lack of top management commitment (B1)	
3	Insufficient human resources for green practices (B3) Lack of employees’ capability to identify environmental opportunities (B4)	Third
4	Lack of rules and regulations for green practices (B15)	Second
	Lack of government subsidy(B11)	
	Lack of capital/resources (B10)	
	Lack of knowledge related to green innovation and practices (B5)	
5	Lack of enforceable laws regarding returned and recycled products (B17)	First

**Table 7:** Comparison of green innovation barriers in different countries.

Ranking	Pakistan	India	Saudi Arabia	Hong Kong
1	Lack of enforceable laws regarding returned and recycled products	Lack of financial support	Implementation of environmental policies	Additional cost due to green innovation
2	Lack of rules and regulations for green practices	Lack of technological capability	Lack of commitment	Possible delays due to green requirement
3	Lack of collaboration with government and environmental institutions	Lack of required infrastructure	Unwillingness to switch to green practices	Limited availability and reliability of green suppliers
4	Lack of capital/resources	Lack of skilled human resources	Lack of government policies to upgrade green technology	Limited knowledge on green technology and materials
5	Lack of government subsidy	Insufficient knowledge	Lack of R&D capacity	Unachievable specification requirements

## Data Availability

The data that support the findings of this study are available on reasonable request from the corresponding author.

## Appendix A

**Table A1:** Level partitions (Iteration 1).

Serial	Reachability Set	Antecedent Set	Intersection Set	Level
1	1, 2, 3, 4, 5, 6, 7, 8, 9, 10, 11, 12, 13, 14, 16, 18	1, 2, 3, 4, 5, 6, 8, 9, 10, 11, 12, 13, 14, 15, 16, 17, 18	1, 2, 3, 4, 5, 6, 8, 9, 10, 11, 12, 13, 14, 15, 16, 18	
2	1, 2, 4, 6, 7, 8, 9, 12, 13, 14, 16, 18	1, 2, 3, 4, 5, 6, 8, 9, 10, 11, 12, 13, 14, 15, 16, 17, 18	1, 2, 4, 6, 8, 9, 12, 13, 14, 16, 18	
3	1, 2, 3, 4, 5, 6, 7, 8, 9, 11, 12, 13, 14, 15, 16, 18	1, 3, 4, 5, 6, 8, 9, 10, 11, 12, 13, 14, 15, 16, 17, 18	1, 2, 3, 4, 5, 6, 8, 9, 11, 12, 13, 14, 15, 16, 18	
4	1, 2, 3, 4, 6, 7, 8, 9, 10, 12, 13, 14, 16, 18	1, 2, 3, 4, 5, 6, 8, 9, 10, 11, 13, 14, 15, 16, 17, 18	1, 2, 3, 4, 6, 8, 9, 10, 12, 13, 14, 16, 18	
5	1, 2, 3, 4, 5, 6, 7, 8, 9, 10, 11, 12, 13, 14, 15, 16, 17, 18	1, 3, 5, 6, 8, 9, 10, 11, 12, 13, 14, 15, 16, 17, 18	1, 3, 5, 6, 8, 9, 10, 11, 12, 13, 14, 15, 16, 17, 18	
6	1, 2, 3, 4, 5, 6, 7, 8, 9, 10, 11, 12, 13, 14, 16, 18	1, 2, 3, 4, 5, 6, 8, 9, 10, 11, 12, 13, 14, 15, 16, 17, 18	1, 2, 3, 4, 5, 6, 8, 9, 10, 11, 12, 13, 14, 15, 16, 18	
7	7	1, 2, 3, 4, 5, 6, 7, 8, 9, 10, 11, 12, 13, 14, 15, 16, 17, 18	7	1st
8	1, 2, 3, 4, 5, 6, 7, 8, 9, 10, 11, 12, 13, 14, 16, 18	1, 2, 3, 4, 5, 6, 8, 9, 10, 11, 12, 13, 14, 15, 16, 17, 18	1, 2, 3, 4, 5, 6, 8, 9, 10, 11, 12, 13, 14, 16, 18	
9	1, 2, 3, 4, 5, 6, 7, 8, 9, 10, 11, 12, 13, 14, 16, 18	1, 2, 3, 4, 5, 6, 8, 9, 10, 11, 12, 13, 14, 15, 16, 17, 18	1, 2, 3, 4, 5, 6, 8, 9, 10, 11, 12, 13, 14, 16, 18	
10	1, 2, 3, 4, 5, 6, 7, 8, 9, 10, 11, 12, 13, 14, 15, 16, 17, 18	1, 4, 5, 6, 8, 9, 10, 11, 12, 13, 14, 15, 16, 17, 18	1, 4, 5, 6, 8, 9, 10, 11, 12, 13, 14, 15, 16, 17, 18	
11	1, 2, 3, 4, 5, 6, 7, 8, 9, 10, 11, 12, 13, 14, 15, 16, 18	1, 3, 5, 6, 8, 9, 10, 11, 12, 13, 14, 15, 16, 17, 18	1, 3, 5, 6, 8, 9, 10, 11, 12, 13, 14, 15, 16, 18	
12	1, 2, 3, 5, 6, 7, 8, 9, 10, 11, 12, 13, 14, 15, 16, 17, 18	1, 2, 3, 4, 5, 6, 8, 9, 10, 11, 12, 13, 14, 15, 16, 17, 18	1, 2, 3, 4, 5, 6, 8, 9, 10, 11, 12, 13, 14, 15, 16, 17, 18	
13	1, 2, 3, 4, 5, 6, 7, 8, 9, 10, 11, 12, 13, 14, 15, 16, 17, 18	1, 2, 3, 4, 5, 6, 8, 9, 10, 11, 12, 13, 14, 15, 16, 17, 18	1, 2, 3, 4, 5, 6, 8, 9, 10, 11, 12, 13, 14, 15, 16, 17, 18	
14	1, 2, 3, 4, 5, 6, 7, 8, 9, 10, 11, 12, 13, 14, 15, 16, 17, 18	1, 2, 3, 4, 5, 6, 8, 9, 10, 11, 12, 13, 14, 15, 16, 17, 18	1, 2, 3, 4, 5, 6, 8, 9, 10, 11, 12, 13, 14, 15, 16, 17, 18	
15	1, 2, 3, 4, 5, 6, 7, 8, 9, 10, 11, 12, 13, 14, 15, 16, 17, 18	3, 5, 10, 11, 12, 13, 14, 15, 16, 17, 18	3, 5, 10, 11, 12, 13, 14, 15, 16, 17, 18	
16	1, 2, 3, 4, 5, 6, 7, 8, 9, 10, 11, 12, 13, 14, 15, 16, 17, 18	1, 2, 3, 4, 5, 6, 8, 9, 10, 11, 12, 13, 14, 15, 16, 17, 18	1, 2, 3, 4, 5, 6, 8, 9, 10, 11, 12, 13, 14, 15, 16, 17, 18	
17	1, 2, 3, 4, 5, 6, 7, 8, 9, 10, 11, 12, 13, 14, 15, 16, 17, 18	5, 10, 12, 13, 14, 15, 16, 17, 18	5, 10, 12, 13, 14, 15, 16, 17, 18	
18	1, 2, 3, 4, 5, 6, 7, 8, 9, 10, 11, 12, 13, 14, 15, 16, 17, 18	1, 2, 3, 4, 5, 6, 8, 9, 10, 11, 12, 13, 14, 15, 16, 17, 18	1, 2, 3, 4, 5, 6, 8, 9, 10, 11, 12, 13, 14, 15, 16, 17, 18	

**Table A2:** Level partitioning (Level 2).

Serial	Reachability Set	Antecedent Set	Intersection Set	Level
1	1, 2, 3, 4, 5, 6, 8, 9, 10, 11, 12, 13, 14, 16, 18	1, 2, 3, 4, 5, 6, 8, 9, 10, 11, 12, 13, 14, 15, 16, 17, 18	1, 2, 3, 4, 5, 6, 8, 9, 10, 11, 12, 13, 14, 16, 18	2nd
2	1, 2, 4, 6, 8, 9, 12, 13, 14, 16, 18	1, 2, 3, 4, 5, 6, 8, 9, 10, 11, 12, 13, 14, 15, 16, 17, 18	1, 2, 4, 6, 8, 9, 12, 13, 14, 16, 18	2nd
3	1, 2, 3, 4, 5, 6, 8, 9, 11, 12, 13, 14, 15, 16, 18	1, 3, 4, 5, 6, 8, 9, 10, 11, 12, 13, 14, 15, 16, 17, 18	1, 3, 4, 5, 6, 8, 9, 11, 12, 13, 14, 15, 16, 18	
4	1, 2, 3, 4, 6, 8, 9, 10, 12, 13, 14, 16, 18	1, 2, 3, 4, 5, 6, 8, 9, 10, 11, 13, 14, 15, 16, 17, 18	1, 2, 3, 4, 6, 8, 9, 10, 13, 14, 16, 18	
5	1, 2, 3, 4, 5, 6, 8, 9, 10, 11, 12, 13, 14, 15, 16, 17, 18	1, 3, 5, 6, 8, 9, 10, 11, 12, 13, 14, 15, 16, 17, 18	1, 3, 5, 6, 8, 9, 10, 11, 12, 13, 14, 15, 16, 17, 18	
6	1, 2, 3, 4, 5, 6, 8, 9, 10, 11, 12, 13, 14, 16, 18	1, 2, 3, 4, 5, 6, 8, 9, 10, 11, 12, 13, 14, 15, 16, 17, 18	1, 2, 3, 4, 5, 6, 8, 9, 10, 11, 12, 13, 14, 16, 18	2nd
8	1, 2, 3, 4, 5, 6, 8, 9, 10, 11, 12, 13, 14, 16, 18	1, 2, 3, 4, 5, 6, 8, 9, 10, 11, 12, 13, 14, 15, 16, 17, 18	1, 2, 3, 4, 5, 6, 8, 9, 10, 11, 12, 13, 14, 16, 18	2nd
9	1, 2, 3, 4, 5, 6, 8, 9, 10, 11, 12, 13, 14, 16, 18	1, 2, 3, 4, 5, 6, 8, 9, 10, 11, 12, 13, 14, 15, 16, 17, 18	1, 2, 3, 4, 5, 6, 8, 9, 10, 11, 12, 13, 14, 16, 18	2nd
10	1, 2, 3, 4, 5, 6, 8, 9, 10, 11, 12, 13, 14, 15, 16, 17, 18	1, 4, 5, 6, 8, 9, 10, 11, 12, 13, 14, 15, 16, 17, 18	1, 4, 5, 6, 8, 9, 10, 11, 12, 13, 14, 15, 16, 17, 18	
11	1, 2, 3, 4, 5, 6, 8, 9, 10, 11, 12, 13, 14, 15, 16, 18	1, 3, 5, 6, 8, 9, 10, 11, 12, 13, 14, 15, 16, 17, 18	1, 3, 5, 6, 8, 9, 10, 11, 12, 13, 14, 15, 16, 18	
12	1, 2, 3, 5, 6, 8, 9, 10, 11, 12, 13, 14, 15, 16, 17, 18	1, 2, 3, 4, 5, 6, 8, 9, 10, 11, 12, 13, 14, 15, 16, 17, 18	1, 2, 3, 5, 6, 8, 9, 10, 11, 12, 13, 14, 15, 16, 17, 18	2nd
13	1, 2, 3, 4, 5, 6, 8, 9, 10, 11, 12, 13, 14, 15, 16, 17, 18	1, 2, 3, 4, 5, 6, 8, 9, 10, 11, 12, 13, 14, 15, 16, 17, 18	1, 2, 3, 4, 5, 6, 8, 9, 10, 11, 12, 13, 14, 15, 16, 17, 18	2nd
14	1, 2, 3, 4, 5, 6, 8, 9, 10, 11, 12, 13, 14, 15, 16, 17, 18	1, 2, 3, 4, 5, 6, 8, 9, 10, 11, 12, 13, 14, 15, 16, 17, 18	1, 2, 3, 4, 5, 6, 8, 9, 10, 11, 12, 13, 14, 15, 16, 17, 18	2nd
15	1, 2, 3, 4, 5, 6, 8, 9, 10, 11, 12, 13, 14, 15, 16, 17, 18	3, 5, 10, 11, 12, 13, 14, 15, 16, 17, 18	3, 5, 10, 11, 12, 13, 14, 15, 16, 17, 18	
16	1, 2, 3, 4, 5, 6, 8, 9, 10, 11, 12, 13, 14, 15, 16, 17, 18	1, 2, 3, 4, 5, 6, 8, 9, 10, 11, 12, 13, 14, 15, 16, 17, 18	1, 2, 3, 4, 5, 6, 8, 9, 10, 11, 12, 13, 14, 15, 16, 17, 18	2nd
17	1, 2, 3, 4, 5, 6, 8, 9, 10, 11, 12, 13, 14, 15, 16, 17, 18	5, 10, 12, 13, 14, 15, 16, 17, 18	5, 10, 12, 13, 14, 15, 16, 17, 18	
18	1, 2, 3, 4, 5, 6, 8, 9, 10, 11, 12, 13, 14, 15, 16, 17, 18	1, 2, 3, 4, 5, 6, 8, 9, 10, 11, 12, 13, 14, 15, 16, 17, 18	1, 2, 3, 4, 5, 6, 8, 9, 10, 11, 12, 13, 14, 15, 16, 17, 18	2nd

**Table A3:** Level partitioning (Level 3).

Serial	Reachability Set	Antecedent Set	Intersection Set	Level
3	3, 4, 5, 11, 15	3, 4, 5, 10, 11, 15, 17	3, 4, 5, 11, 15	3rd
4	3, 4, 10	3, 4, 5, 10, 11, 15, 17	3, 4, 10	3rd
5	3, 4, 5, 10, 11, 15, 17	3, 5, 10, 11, 15, 17	3, 5, 10, 11, 15, 17	
10	3, 4, 5, 10, 11, 15, 17	4, 5, 10, 11, 15, 17	4, 5, 10, 11, 15, 17	
11	3, 4, 5, 10, 11, 15	3, 5, 10, 11, 15, 17	3, 5, 10, 11, 15	
15	3, 4, 5, 10, 11, 15, 17	3, 5, 10, 11, 15, 17	3, 5, 10, 11, 15, 17	
17	3, 4, 5, 10, 11, 15, 17	5, 10, 15, 17	5, 10, 15, 17	

**Table A4:** Level partitioning (Level 4).

Serial	Reachability Set	Antecedent Set	Intersection Set	Level
5	5, 10, 11, 15, 17	5, 10, 11, 15, 17	5, 10, 11, 15, 17	4th
10	5, 10, 11, 15, 17	5, 10, 11, 15, 17	5, 10, 11, 15, 17	4th
11	5, 10, 11, 15	5, 10, 11, 15, 17	5, 10, 11, 15	4th
15	5, 10, 11, 15, 17	5, 10, 11, 15, 17	5, 10, 11, 15, 17	4th
17	5, 10, 11, 15, 17	5, 10, 15, 17	5, 10, 15, 17	

**Table A5:** Level partitioning (Level 5).

Serial	Reachability Set	Antecedent Set	Intersection Set	Level
17	17	17	17	5th
